# Association of accelerated long-term forgetting and senescence-related blood-borne factors in asymptomatic individuals from families with autosomal dominant Alzheimer’s disease

**DOI:** 10.1186/s13195-021-00845-0

**Published:** 2021-05-27

**Authors:** Jianwei Yang, Chaojun Kong, Longfei Jia, Tingting Li, Meina Quan, Yan Li, Diyang Lyu, Fangyu Li, Hongmei Jin, Ying Li, Qigeng Wang, Jianping Jia

**Affiliations:** 1 Innovation Center for Neurological Disorders and Department of Neurology, Xuanwu Hospital, Capital Medical University, National Clinical Research Center for Geriatric Diseases, 45 Changchun St, Beijing, China; 2 Beijing Key Laboratory of Geriatric Cognitive Disorders, Beijing, China; 3 Clinical Center for Neurodegenerative Disease and Memory Impairment, Capital Medical University, Beijing, China; 4 Center of Alzheimer’s Disease, Beijing Institute of Brain Disorders, Collaborative Innovation Center for Brain Disorders, Capital Medical University, Beijing, China

**Keywords:** Alzheimer’s disease, Accelerated long-term forgetting, Blood-borne factors, Senescence, Biomarkers

## Abstract

**Background:**

Accelerated long-term forgetting has been identified in preclinical Alzheimer’s disease (AD) and is attributed to a selective impairment of memory consolidation in which the hippocampus plays a key role. As blood may contain multiple senescence-related factors that involved in neurogenesis and synaptic plasticity in the hippocampus, we tested whether there is an association between blood-borne factors and accelerated long-term forgetting in asymptomatic individuals from families with autosomal dominant AD (ADAD).

**Methods:**

We analyzed data of 39 asymptomatic participants (*n* = 18 ADAD mutation carriers, *n* = 21 non-carriers) from the Chinese Familial Alzheimer’s Disease Network (CFAN) study. Long-term forgetting rates were calculated based on recall or recognition of two materials (word list and complex figure) at three delays comprising immediate, 30 min, and 7 days. Peripheral blood concentrations of candidate pro-aging factors (CC chemokine ligand 11 [CCL11] and monocyte chemotactic protein 1 [MCP1]) and rejuvenation factors (growth differentiation factor 11 [GDF11], thrombospondin-4 [THBS4], and secreted protein acidic and rich in cysteine like 1 [SPARCL1]) were evaluated in all participants.

**Results:**

Despite normal performance on standard 30-min delayed testing, mutation carriers exhibited accelerated forgetting of verbal and visual material over 7 days in comparison with matched non-carriers. In the whole sample, lower plasma THBS4 was associated with accelerated long-term forgetting in list recall (β = −0.46, *p* = 0.002), figure recall (β = −0.44, *p *= 0.004), and list recognition (β = −0.37, *p* = 0.010). Additionally, higher plasma GDF11 and CCL11 were both associated with accelerated long-term forgetting (GDF11 versus figure recall: β = 0.39, *p* = 0.007; CCL11 versus list recognition: β = 0.44, *p* = 0.002).

**Conclusions:**

Accelerated long-term forgetting is a cognitive feature of presymptomatic AD. Senescence-related blood-borne factors, especially THBS4, GDF11, and CCL11, may be promising biomarkers for the prediction of accelerated long-term forgetting.

**Supplementary Information:**

The online version contains supplementary material available at 10.1186/s13195-021-00845-0.

## Background

Alzheimer’s disease (AD) is a clear and present concern to public health in China and internationally and has a profound socio-economic impact [1]. Early identification of AD has become a priority in research, as it may open the door to preventive approaches [2]. The fact that AD pathological changes develop decades before visible cognitive impairments might reflect that the sensitivities of the cognitive assays currently used are not sufficient enough [3, 4]. In this context, increasing evidence has suggested that accelerated long-term forgetting would be a subtle cognitive impairment in presymptomatic autosomal dominant AD (ADAD) mutation carriers [5, 6]. This memory impairment was thought to be attributed to a failure of memory consolidation process, which was primarily governed by the medial temporal lobe (MTL), especially the hippocampus [7].

A flurry of studies has demonstrated that specific factors are present in the blood that directly affect neurogenesis and synaptic plasticity in the hippocampus and thus regulate memory and cognitive function [8–12]. For example, initial studies in mouse models have identified pro-aging factors (monocyte chemotactic protein 1 [MCP1] and CC chemokine ligand 11 [CCL11]) [8] and rejuvenating factor (growth differentiation factor 11 [GDF11]) [9] that correlated with hippocampal neurogenesis and cognitive function. More recently, both thrombospondin-4 (THBS4) and secreted protein acidic and rich in cysteine like 1 (SPARCL1) were found to represent rejuvenation factors that enhance synaptic connectivity [10]. These results provide support for the idea that senescence-related blood-borne factors would be served as sensitive predictors of cognitive function (specifically hippocampal memory). However, few studies have examined these senescence-related blood-borne factors with respect to subtle cognitive change, especially hippocampal-dependent memory impairments, in the preclinical stage of AD.

The goal of this study was to explore whether blood-borne factors were associated with long-term forgetting rates in asymptomatic carriers and non-carriers of ADAD mutations. We expected to find that higher pro-aging factors or lower rejuvenation factors that regulate hippocampal neurogenesis and synaptic plasticity would be associated with accelerated long-term forgetting in the early stage of AD.

## Methods

### Participants

All participants in this study were recruited from the Chinese Familial Alzheimer’s Disease Network (CFAN), which is a multicenter, longitudinal cohort used to collect the hereditary and clinical profile of familial AD [13]. Eighteen participants were mutation carriers with a known causative mutation of AD, including 11 carrying *presenilin 1* (*PSEN1*) mutation, 4 carrying *amyloid precursor protein* (*APP*) mutation, and 3 carrying *presenilin 2* (*PSEN2*) mutation. Twenty-one were non-carrier family members. The estimated proximity to onset for mutation carriers of ADAD was calculated as the age of the participant at assessment minus the age of the parent at symptom onset [14]. All participants were blind to their genetic status, in accordance with cultural norms and ethical regulations in this community. Inclusion criteria were Clinical Dementia Rating (CDR) global score = 0 and Mini-Mental State Examination (MMSE) score ≥ 26. None of the participants reported complaints of anxiety or depressive symptoms. This study was approved by the Medical Research Ethics Committee of Xuanwu Hospital, Capital Medical University. All participants in the study provided written informed consent.

### Standard neuropsychological testing

Clinical disease severity was rated using the CDR scale, which incorporates information from both participant and informant on day-to-day cognition [15]. The MMSE [16] and Montreal Cognitive Assessment (MoCA) [17] were used to evaluate global cognitive function. Self-report of subject cognitive concerns was measured with the participant (MyCog) version of the Subjective Cognitive Decline Questionnaire (SCD-Q), which quantifies perceived subjective cognitive decline over the last 2 years [18]. Subscores for memory (items 1–11), language (items 12–17), and executive (items 18–24) functions were also computed.

### Assessment of long-term forgetting rates

The WHO-University of California Los Angeles-Auditory Verbal Learning test (WHO-UCLA-AVLT) was used to assess verbal learning, recall, and recognition performances [19]. This version of the auditory verbal learning test consists of a list of 15 non-associated words that is presented orally. During this test, participants had to learn the material to a minimum required accuracy of 80% at free recall over a minimum of 3 and a maximum of 10 trials. The Rey-Osterrieth Complex Figure (ROCF) test was used to examine visuospatial copy and recall [20]. During this test, participants were asked to copy the figure as accurately as possible and were instructed that they would later be asked to draw it from memory. Both the figure and copy were then removed from view and participants were asked to draw the figure again from memory.

At 30 min and 7 days later, participants were once again asked to recall the word list and to draw the figure from memory in as much detail as they could remember. For the word list, a recognition test was also performed during which the 15 target words were presented among 15 distracters. There was no recognition for the visual memory test. Participants were not informed about the 7-day follow-up assessment, which was conducted over the phone. This ensures that participants would not be motivated to rehearse. The 7-day follow-up figure assessments were returned by mail. The long-term forgetting rate is defined as the percentage of information lost between 30-min and 7-day delays, as follows: (1 − performance at 7-day delay/performance at 30-min delay) × 100 [21].

### Blood collection and analysis

Non-fasting venous blood for plasma analyses was collected in EDTA-containing tubes, which were then centrifuged at 2000×*g* for 15 min at 4°C. For serum analyses, blood samples were collected in tubes without anticoagulant and centrifuged after removal of clots of blood at room temperature for a half hour. EDTA plasma and serum samples were stored in single-use aliquots at −80°C until biochemical analysis.

Plasma CCL11, MCP1, GDF11, THBS4, and serum SPARCL1 concentrations were detected using enzyme-linked immunosorbent assay (ELISA; Supplementary Table 1). All analyses were performed in duplicate, according to the manufacturer’s published protocols. Final data were examined for extreme outliers, and samples with >50× the upper interquartile range were also excluded (*n* = 1 on MCP1).

### Statistical analyses

Group differences in participant characteristics were assessed using Mann–Whitney U tests for continuous and chi-square test for categorical variables. Differences in long-term memory performance were compared between groups using multivariate linear regression, adjusted for age, sex, and education. Multiple comparisons were *p* corrected for false discovery rate (FDR). Receiver operating characteristic (ROC) curve analysis was used to determine the ability of long-term forgetting rates to discriminate between mutation carriers and non-carriers. Spearman’s correlations were used to test associations among long-term forgetting rates, estimated proximity to onset, and subject cognitive concerns in mutation carriers. To assess the individual contribution of each blood-borne factor on the prediction of accelerated long-term forgetting, multivariate analyses were conducted using stepwise linear regression models with long-term forgetting rates as dependent variables and the blood-borne factors as independent factors. Models were adjusted for age, sex, and education. The statistical analyses were performed with IBM SPSS software. All tests were two-sided, and *p* values less than 0.05 were considered statistically significant.

## Results

### Sample characteristics

Demographic, clinical, and neuropsychological characteristics of the sample are presented in Table 1. As anticipated, mutation carriers did not differ from non-carriers in basic demographic variables, such as age, sex, and education attainment. There were no significant differences regarding the global level of cognition and subjective cognitive ratings between carriers and non-carriers. There was no difference in plasma concentrations of CCL11, MCP1, GDF11, and THBS4 between mutation carriers and non-carriers. The between-group difference of serum SPARCL1 concentration was not significant, either.

**Table 1:** Demographic, clinical, and neuropsychological characteristics of study participants

Variable	Mutation carriers (n = 18)	Non-carriers (n = 21)	***p*** value
**Demographic data**			
Female, n (%)	8 (44.4)	11 (52.4)	0.751
Age, y	38.0 (35.8–43.0)	35.0 (33.0–48.0)	0.282
Estimated proximity to onset, y	11.0 (7.8–15.5)	NA	
Education, y	14.0 (11.0–15.3)	16.0 (11.5–16.5)	0.094
**Cognitive level (global)**			
MMSE score	29.5 (29.0–30.0)	30.0 (29.0–30.0)	0.512
MoCA score	28.0 (27.0–28.0)	27.0 (27.0–28.5)	0.606
**SCD-Q MyCog**			
Total score	6.0 (2.0–9.3)	2.0 (0.5–7.0)	0.272
Memory subscore	4.0 (1.8–6.0)	2.0 (0–4.5)	0.185
Language subscore	1.0 (0–2.0)	0 (0–1.0)	0.262
Executive subscore	1.0 (0–1.5)	0 (0–1.0)	0.150
**Senescence-related factor**			
CCL11, pg/ml	77.4 (64.1–92.4)	76.9 (51.2–90.6)	0.455
MCP1, pg/ml	179.0 (137.3–212.3)	149.4 (122.5–194.2)	0.537
GDF11, pg/ml	87.9 (52.6–118.2)	72.6 (42.4–91.2)	0.673
THBS4, ng/ml	120.7 (85.2–161.3)	159.4 (117.5–217.5)	0.530
SPARCL1, ng/ml	5.4 (3.9–7.3)	5.2 (4.1–6.8)	0.749

For continuous variables, data are shown as median (IQR), and the Mann–Whitney U test was used for group comparison; for categorical variables, the chi-square test was used for group comparison

*Abbreviations*: *CCL11* CC chemokine ligand 11, *GDF11* growth differentiation factor 11, *MCP1* monocyte chemotactic protein 1, *MMSE* Mini-Mental State Examination, *MoCA* Montreal Cognitive Assessment, *NA* not applicable, *SCD-Q* Subjective Cognitive Decline Questionnaire, *SPARCL1* secreted protein acidic and rich in cysteine like 1, *THBS4* thrombospondin-4

### Differences between groups in long-term memory performance

Age-, sex-, and education-adjusted comparisons of long-term memory performance revealed significantly poorer retention of material at 7-day recall scores for list and figure in the mutation carriers compared to non-carriers (Table 2). Mutation carriers also did worse than non-carriers on 7-day list recognition. No significant group differences were found for the initial score and the number of learning trials. In addition, the recall and recognition score at 30 min for list, as well as the recall score at 30 min for figure, did not differ between the two groups.

**Table 2:** Group differences in long-term memory performance

	Mutation carriers	Non-carriers	***p*** value	Difference in mean	95% CI
**Learning**					
List learning trials	4.0 (4.0–5.0)	4.0 (4.0–5.0)	0.981	−0.01	−0.71 to 0.69
List learning score	80.0 (80.0–86.7)	86.7 (80.0–86.7)	0.570	−1.38	−4.55 to 1.78
Figure learning score	72.9 (63.2–80.6)	69.4 (60.4–76.4)	0.579	4.95	−2.61 to 12.51
**30-min retention**					
List 30-min recall	73.3 (66.7–80.0)	73.3 (70.0–80.0)	0.681	−3.46	−9.15 to 2.25
Figure 30-min recall	69.4 (56.9–79.5)	66.7 (56.9–72.2)	0.418	3.36	−4.96 to 11.67
List 30-min recognition	90.0 (73.3–93.3)	93.3 (83.3–93.3)	0.441	−3.64	−10.57 to 3.29
**7-day retention**					
List 7-day recall	53.3 (40.0–60.0)	66.7 (60.0–80.0)	<0.001	−15.34	−22.23 to −8.46
Figure 7-day recall	38.9 (33.3–52.8)	58.3 (47.9–60.4)	0.002	−12.31	−19.20 to −5.42
List 7-day recognition	70.0 (53.3–80.0)	80.0 (73.3–90.0)	0.006	−11.52	−19.50 to −3.53
**Long-term forgetting rate**					
Forgetting rate in list recall	26.1 (18.2–34.1)	9.1 (0.0–14.1)	<0.001	17.26	11.21 to 23.32
Forgetting rate in figure recall	36.3 (28.4–48.1)	10.5 (7.1–20.4)	<0.001	22.30	14.57 to 30.03
Forgetting rate in list recognition	20.7 (14.3–28.9)	7.7 (0.0–18.3)	0.003	10.07	3.66 to 16.48

Data are shown as medians (IQR). Multivariate linear regression adjusted for age, sex, and education was used to compare the between-group scores

### Diagnostic value of long-term forgetting rates

**Fig. 1:**
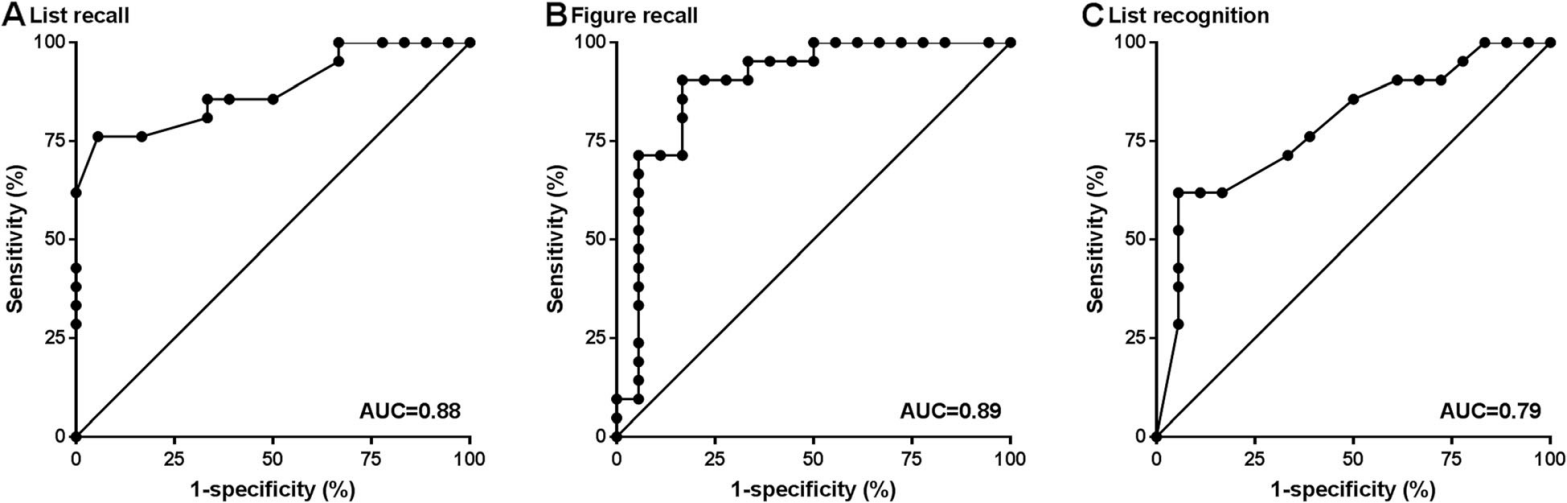
ROC curves for long-term forgetting rates as a discriminator between preclinical mutation carriers and non-carriers. ROC curves are shown for long-term forgetting rates for **a** list recall, **b** figure recall, and **c** list recognition

ROC analysis for discrimination between mutation carriers and non-carriers for long-term forgetting rates revealed an AUC of 0.881 (95% CI 0.774–0.988) for list recall, 0.894 (0.783–1.000) for figure recall, and 0.790 (0.645–0.934) for list recognition (Fig. 1).

### Association of blood-borne factors with long-term forgetting rates

There was a significant association between blood-borne factors and long-term forgetting rates in the whole sample, adjusted for age, sex, and education (Table 3). Lower plasma THBS4 was associated with accelerated long-term forgetting in list recall (β = −0.46, *p* = 0.002), figure recall (β = −0.44, *p* = 0.004), and list recognition (β = −0.37, *p* = 0.010). In addition, there were significant associations of higher plasma GDF11 with accelerated forgetting in figure recall (β = 0.39, *p* = 0.007) and higher plasma CCL11 with accelerated forgetting in list recognition (β = 0.44, *p* = 0.002).

**Table 3:** Association of blood-borne factors with long-term forgetting rates

Dependent variables		Independent factors	B	B SE	β	***p*** value	R^**2**^
**The whole sample**							
Forgetting rate in list recall	Step 1	Age	0.84	0.24	0.51	0.001	0.24
	Step 2	Age	0.97	0.23	0.59	< 0.001	0.33
		THBS4	−0.07	0.03	−0.34	0.020	
	Step 3	Age	1.14	0.22	0.69	< 0.001	0.43
		THBS4	−0.10	0.03	−0.46	0.002	
		Sex	−10.53	3.93	−0.36	0.011	
Forgetting rate in figure recall	Step 1	GDF11	0.17	0.06	0.44	0.006	0.17
	Step 2	GDF11	0.15	0.05	0.38	0.011	0.28
		THBS4	−0.08	0.03	−0.36	0.016	
	Step 3	GDF11	0.15	0.05	0.39	0.007	0.34
		THBS4	−0.10	0.03	−0.44	0.004	
		Sex	−8.94	4.35	−0.29	0.048	
Forgetting rate in list recognition	Step 1	CCL11	0.20	0.06	0.46	0.003	0.19
	Step 2	CCL11	0.20	0.06	0.47	0.002	0.30
		THBS4	−0.05	0.02	−0.30	0.042	
	Step 3	CCL11	0.19	0.06	0.44	0.002	0.35
		THBS4	−0.06	0.02	−0.37	0.010	
		Age	0.42	0.18	0.32	0.025	
**Mutation carriers**							
Forgetting rate in list recall		Age	1.16	0.33	0.67	0.003	0.41
Forgetting rate in figure recall		GDF11	0.17	0.06	0.59	0.012	0.31
**Non-carriers**							
Forgetting rate in list recall		MCP1	0.10	0.03	0.66	0.001	0.41
Forgetting rate in figure recall		THBS4	−0.05	0.02	−0.44	0.046	0.15
Forgetting rate in list recognition		CCL11	0.20	0.08	0.49	0.025	0.20

Results of multivariate linear regression analysis (stepwise) with the age, sex, education, and blood-borne factors as independent factors, and the long-term forgetting rates as dependent variables. Only statistically significant associations are listed (p  <  0.05)

*Abbreviations*: *CCL11* CC chemokine ligand 11, *GDF11* growth differentiation factor 11, *MCP1* monocyte chemotactic protein 1, *THBS4* thrombospondin-4

In mutation carriers, there was a consistent association between higher plasma GDF11 and accelerated forgetting in figure recall (β = 0.59, *p* = 0.012), but no evidence of correlations between any other blood-borne factors and long-term forgetting rates.

### Associations between estimated proximity to onset and long-term forgetting rates

**Fig. 2:**
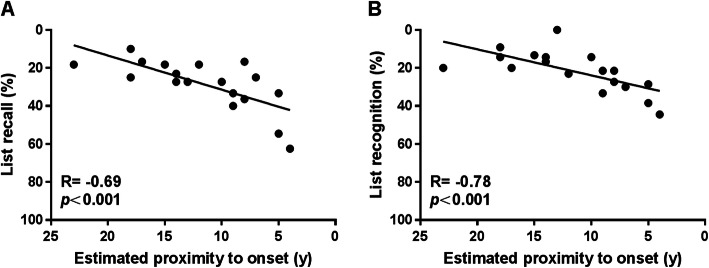
Scatter plots for estimated proximity to onset against long-term forgetting rates in mutation carriers. Association between estimated proximity to onset and **a** forgetting rate in list recall and **b** forgetting rate in list recognition

In mutation carriers, there was an association between estimated proximity to onset and long-term forgetting rates for list recall (R = −0.69, *p* = 0.002; Fig. 2a) and recognition (R = −0.78, *p* < 0.001; Fig. 2b), such that those who were close to clinical onset showed faster forgetting rates. However, no significant relationships were found for the figure recall (R = −0.44, *p* = 0.071).

### Association between long-term forgetting rates and subject cognitive concerns

In the whole sample and mutation carriers, an association existed between higher total score or memory subscore of SCD-Q MyCog and accelerated long-term forgetting, which reached statistical significance for list recall, but not for figure recall and list recognition (Supplementary Table 2).

## Discussion

The present study added more evidence to existing literature characterizing accelerated long-term forgetting as a subclinical cognitive impairment in ADAD mutation carriers. Further, we showed that there is an association between senescence-related blood-borne factors and accelerated long-term forgetting in pooled participants with mutation carriers and non-carriers. To our knowledge, this study is the first that attempt to determine the association of blood-borne factors and accelerated long-term forgetting in asymptomatic individuals. Our findings revealed the promise of blood-borne factors as potential biomarkers for the prediction of accelerated long-term forgetting.

In this study, we have demonstrated that accelerated long-term forgetting is an early feature of presymptomatic ADAD mutation carriers, who were several years before their predicted age at symptom onset. Our finding was consistent with those reported in previous studies that accelerated long-term forgetting is present in presymptomatic ADAD mutation carriers, and might provide a means of discriminating presymptomatic AD from controls [5, 6]. In addition, accelerated long-term forgetting was associated with greater subjective cognitive concerns in mutation carriers. This association is also consistent with findings from the previous study [5]. As for sporadic AD, accelerated long-term forgetting was also found in asymptomatic *APOE* ε4 carriers with a mean age well below their predicted age at symptom onset [22, 23]. More recently, a longitudinal study showed that accelerated long-term forgetting over 4 weeks could predict cognitive decline in healthy older people [7]. Together, these results highlight the potential of accelerated long-term forgetting in screening for patient candidates and guiding the selection of outcome measures in future preclinical trials.

The molecular mechanisms underlying accelerated long-term forgetting are not fully understood. The pattern of relatively intact retention over short delays, coupled with poor retention after longer delays, is suggestive of a selective impairment of memory consolidation [24]. Memory consolidation is the process that transforms the labile memory traces into stable long-term memories, with the hippocampus and MTL cortices known to play an important part [25]. Therefore, it is currently believed that impaired memory consolidation caused by hippocampal dysfunction might produce accelerated long-term forgetting. For example, a recent study has shown that both seizures and interictal spikes in the hippocampus during sleep impair long-term memory consolidation, supporting the notion that hippocampal dysfunction causes accelerated long-term forgetting [26].

Hippocampal dysfunction has been used to explain the phenomenon of accelerated long-term forgetting, as mentioned above. Thus, blood-borne factors that contribute to neurogenesis and synaptic plasticity in the hippocampus could provide useful biomarkers for accelerated long-term forgetting. In this study, lower plasma THBS4 was significantly associated with accelerated long-term forgetting. Thrombospondins, including THBS 1-5, are large secreted extracellular matrix proteins that mediate cell–cell and cell–matrix interactions [27]. As members of synaptic organizers, thrombospondins have been previously identified as possible synaptogenic factors in the central nervous system [28, 29]. In a recent in vitro study, THBS4 was found to directly stimulate dendritogenesis and synapse formation in cultured neurons [10]. Given the fact that it may be possible for THBS4 to be transported from the blood into the brain parenchyma and directly activate synapse formation and function [10], it is reasonable to assume that reductions in circulating THBS4 levels might contribute to decrease synaptic connectivity and cognitive impairments. Thus, lower levels of plasma THBS4 in relation to accelerated long-term forgetting in our study are to be expected.

Additionally, we found that higher plasma GDF11, a secreted member of the transforming growth factor β (TGF-β) superfamily, is associated with accelerated long-term forgetting. This is an unexpected finding since GDF11 may enhance neurogenesis in the adult brain [9] and reduced AD pathology has been observed in association with increasing plasma GDF11 concentrations in AD animal models [30]. However, the role of GDF11 in cognition remains controversial. Another study suggested that plasma GDF11 may not exert a protective effect on cognitive function in patients with age-related cognitive impairment [31]. Actually, previous studies have reported either a beneficial or detrimental role of GDF11 in age-related dysfunction [32]. Specifically, some studies have suggested that reduced GDF11 blood levels are responsible for age-related cardiac hypertrophy [33] and skeletal muscle dysfunction [34], while others have shown that increasing levels of GDF11 can induce skeletal and cardiac muscle wasting [35, 36]. In addition, the concentration of GDF11 in plasma was determined using an antibody-based method in our study, which may be thwarted by insufficient specificity due to the high homology between GDF11 and myostatin (MSTN/GDF8) [37]. Accumulating evidence suggests that these two homologous proteins can have opposite effects on biological processes, such as neurogenesis, in the brain [38]. Thus, further studies are required to determine whether and how GDF11 in the blood may influence cognitive function and memory performance.

CCL11, also known as eotaxin-1, is a chemokine that plays an important role in a variety of inflammatory conditions [39]. Although traditionally identified as an eosinophil chemoattractant, CCL11 can be secreted by lymphocytes, monocytes, endothelial cells, and other cells [40]. In the present study, higher plasma CCL11 has also been found to be correlated with accelerated long-term forgetting. This finding is consistent with prior studies using animal models of aging demonstrating increased plasma CCL11 correlate with reduced neurogenesis and impaired learning and memory [8]. Previous studies have suggested that circulating concentration of CCL11 is significantly higher in AD patients than in normal controls [41, 42], and age-related increases in CCL11 are negatively associated with performance on measures of global cognition, executive functions, and episodic memory [43–45]. It is worth mentioning that circulating CCL11 can access many regions of the brain and exert negative influences in the central nervous system via transport across the blood–brain barrier [46]. Thus, blood-borne CCL11 may be involved in the process of AD in aging, and peripheral CCL11 concentrations might be able to predict cognitive impairment.

### Limitations

This study is not without limitations. The first is the relatively small sample size in the present study, which may affect the quality of the statistical analysis. As such, validation in future studies with larger sample sizes is needed to confirm our findings. Another important limitation is the absence of longitudinal assessments of the participants, and we failed to infer causality with the presented data. To fully appreciate the dynamic changes of blood-borne factors and accelerated long-term forgetting along the disease continuum, further follow-up of these participants is needed. Finally, some factors cannot be readily controlled, such as practice effect, 7-day testing environment, and participant’s sleep quality. These methodological issues are important to take into account when investigating accelerated long-term forgetting in our future studies.

## Conclusions

In summary, our study suggests that accelerated long-term forgetting, as a hippocampal-dependent memory impairment, is present at the earlier stage in the AD continuum. Senescence-related blood-borne factors that correlated with hippocampal dysfunction, including THBS4, GDF11, and CCL11, may present ideal biomarkers for accelerated long-term forgetting. Our findings underscore the idea that blood-borne factors may provide insights into subtle cognitive decline in presymptomatic AD, which deserves further studies in the next.

## Supplementary Information

**Additional file 1:** Supplementary data.

## Data Availability

The data generated and analyzed in the current study can be made available upon request to the corresponding author and the CFAN committee in order to ensure that the privacy of the CFAN participants is protected.

## Abbreviations

AD: Alzheimer’s disease
ADAD: Autosomal dominant autosomal dominant
APP: Amyloid precursor protein
CCL11: CC chemokine ligand 11
CDR: Clinical Dementia Rating
CFAN: Chinese Familial Alzheimer’s Disease Network
ELISA: Enzyme-linked immunosorbent assay
GDF11: Growth differentiation factor 11
MCP1: Monocyte chemotactic protein 1
MMSE: Mini-Mental State Examination
MoCA: Montreal Cognitive Assessment
PSEN1: Presenilin 1
PSEN2: Presenilin 2
ROCF: Rey-Osterrieth Complex Figure
ROC: Receiver operating characteristic
SCD-Q: Subjective Cognitive Decline Questionnaire
SPARCL1: Secreted protein acidic and rich in cysteine like 1
THBS4: Thrombospondin-4
WHO-UCLA-AVLT: WHO-University of California Los Angeles-Auditory Verbal Learning test
