# Supplementary material for: Association of accelerated long-term forgetting and senescence-related blood-borne factors in asymptomatic individuals from families with autosomal dominant Alzheimer’s disease
Source: Alzheimers Res Ther. 2021 May 27;13:107. doi: 10.1186/s13195-021-00845-0 (PMC8157428; doi:10.1186/s13195-021-00845-0)
Supplement: Supplementary file 1 — Additional file 1: Supplementary data. [file 13195_2021_845_MOESM1_ESM.docx]

**Supplementary Data**

**Table** **1** ELISA kits information

| **ELISA kits** | **Catalog numbers** | **Assay ranges** | **Current data** | **Intra-assay** **CV** |
| --- | --- | --- | --- | --- |
| **CCL11/ Eotaxin-1**  R&D Systems Inc (USA) | DTX00 | 15.6-1000 pg/ml | 37.2-168.8pg/ml | 1.8% |
| **MCP1/CCL11**  R&D Systems Inc (USA) | DCP00 | 31.2-2000 pg/ml | 46.0-355.3 pg/ml | 1.5% |
| **GDF11**  EIAab Science Co. (China) | E0343h | 15.6-1000 pg/ml | 6.6-202.1 pg/ml | 10.2% |
| **THBS4**  Cusabio Life Sciences (China) | CSB-EL023490HU | 7.8-500 ng/ml | 54.5-358.9 ng/ml | 12.2% |
| **SPARCL1**  Abcam (USA) | ab213826 | 46.9-3000 pg/ml | 1.9-8.6 ng/ml | 7.9% |

The current data ranges indicate the lowest and highest values obtained by using corresponding ELISA kits.

*Abbreviations*: *CCL11* C-C chemokine ligand 11, *CV* coefficient of variation, *GDF11* growth differentiation factor 11, *MCP1* monocyte chemotactic protein 1, *SPARCL1* secreted protein acidic and rich in cysteine like 1, *THBS4* thrombospondin-4

**Table 2** Association between long-term forgetting rates and SCD-Q MyCog scores

|  | **Total score** | **Memory subscore** | **Language subscore** | **Executive subscore** |
| --- | --- | --- | --- | --- |
| **Whole sample** |  |  |  |  |
| Forgetting rate in list recall | -0.399 (0.012) | -0.384 (0.017) | -0.248 (0.133) | -0.273 (0.098) |
| Forgetting rate in figure recall | -0.285 (0.078) | -0.308 (0.060) | -0.071 (0.673) | -0.119 (0.478) |
| Forgetting rate in list recognition | -0.344 (0.032) | -0.282 (0.086) | -0.363 (0.025) | -0.361 (0.026) |
| **Mutation carriers** |  |  |  |  |
| Forgetting rate in list recall | -0.496 (0.036) | -0.516 (0.028) | -0.201 (0.424) | -0.301 (0.225) |
| Forgetting rate in figure recall | -0.262 (0.294) | -0.348 (0.156) | 0.088 (0.728) | 0.091 (0.719) |
| Forgetting rate in list recognition | -0.334 (0.176) | -0.182 (0.470) | -0.297 (0.237) | -0.392 (0.108) |
| **Non-carriers** |  |  |  |  |
| Forgetting rate in list recall | -0.317 (0.162) | -0.238 (0.312) | -0.284 (0.224) | -0.138 (0.560) |
| Forgetting rate in figure recall | -0.248 (0.278) | -0.291 (0.213) | -0.012 (0.960) | -0.121 (0.613) |
| Forgetting rate in list recognition | -0.225 (0.327) | -0.242 (0.304) | -0.425 (0.062) | -0.009 (0.679) |
